# Becoming urban: How city life shapes the social structure and genetics of ants

**DOI:** 10.1111/mec.16657

**Published:** 2022-08-25

**Authors:** Milan Janda

**Affiliations:** ^1^ Investigador Conacyt, Escuela Nacional de Estudios Superiores Unidad Morelia Universidad Nacional Autonoma de Mexico Morelia Mexico; ^2^ Department of Zoology, Faculty of Science Palacky University Olomouc Olomouc Czech Republic; ^3^ Institute of Entomology Biology Centre Czech Academy of Sciences Ceske Budejovice Czech Republic

**Keywords:** population genetics, social insects, urban biology

## Abstract

Cities and urban environments can do peculiar things to biodiversity that shares them with us. How cities affect their invited and uninvited inhabitants has become an increasingly important question. More than half of the world's population dwells in urban areas, and these environments will keep expanding considerably. Understanding how this relatively recent, rapid, and pervasive form of landscape modification influences the ecology and evolution of organisms that cannot escape, or may benefit from it, is an emerging field of biology. Although we are aware of how some birds, mammals or plants respond to urban environments, less is known about insects and invertebrates in general. In this issue of Molecular Ecology, Blumenfeld et al. (2022) bring new remarkable insights into how a common ant species adjusts to urban settings across the United States by changing its social structure and behaviour. Using a large‐scale molecular, chemical and behavioural dataset, they document how the odorous house ant Tapinoma sessile differs in its colony organisation and dispersal strategy between rural and urban habitats. In each of the study regions and continent‐wide, rural and urban colonies are genetically and chemically differentiated, suggesting that urban settings act as potent agents of selection and isolation. The novelty and importance of this study are that it documents multiple independent transitions toward the same social organisation and the apparent effect of habitat on the life history of a eusocial insect species.

## 1 INTRODUCTION

Cities and urban environments can do peculiar things to the biodiversity that shares them with us. How cities affect their invited and uninvited inhabitants has become an increasingly important question. More than half the world's population dwells in urban areas, and these environments will continue to expand considerably. Understanding how this relatively recent, rapid and pervasive form of landscape modification influences the ecology and evolution of organisms that cannot escape, or may benefit from it, is an emerging field of biology. Although we are aware of how some birds, mammals or plants respond to urban environments, less is known about insects and invertebrates in general. In this issue of *Molecular Ecology*, Blumenfeld et al. (2022) bring new remarkable insights into how a common ant species adjusts to urban settings across the United States by changing its social structure and behaviour. Using a large‐scale molecular, chemical and behavioural data set, they document how the odorous house ant *Tapinoma sessile* differs in its colony organization and dispersal strategy between rural and urban habitats. In each of the study regions and continent‐wide, rural and urban colonies are genetically and chemically differentiated, suggesting that urban settings act as potent agents of selection and isolation. The novelty and importance of this study are that it documents multiple independent transitions towards the same social organization and the apparent effect of habitat on the life history of a eusocial insect species.

The biology of urban‐dwelling species has become relevant as we are increasingly concerned about organisms with which we share our living space. Human‐altered environments create novel opportunities to study phenotypic plasticity and adaptations to rapid changes. Different species read cities differently but sometimes consistently to such an extent that we can distinguish urban adapters, exploiters or avoiders. While tall city buildings may seem just like another type of rock formation for the pigeons, swifts or kestrels, for others urban settings represent an entirely new environment, creating an intense selection pressure on their lives. Undeniably, most biodiversity cannot cope and will simply disappear from these highly altered environments. Among the survivors, behavioural responses and other adaptations seem to be shared, even among unrelated species. Such adaptations typically involve increased heat tolerance, adjustments to pollutant exposure, shifts in phenology and activity patterns, faster reproduction, reduced dispersal, etc.

We still know surprisingly little about how these ecological and evolutionary changes compare across diverse taxa and regions, their mechanisms and the extent to which they are parallel. Recent calls for implementing a more systematic framework and experimental approaches to evaluate species' evolutionary responses and plasticity stress the importance of rigorous sampling and comparable measures of urban features in ecological and genetic studies (Dunn et al., 2022; Fusco et al., 2021). A promising approach is to focus on how urbanization affects other successful social species. Compared to solitary organisms, eusocial insects provide distinct layers in which their response can be studied. In addition to individual‐based adaptations, we can analyse how social features of colonies adjust to the new environment and how both link to population‐level processes.

Ants are no strangers to human‐altered environments. Some are notoriously (un)popular species and invaders of disturbed habitats, isolated islands or whole continents. Traits which make ants successful invaders are mainly known and often shared across unrelated species. They are linked to reproduction and dispersal strategies such as polygyny (multiple reproductive queens), dependent colony foundation (budding) or parthenogenesis such as thelytoky. Others consist of behavioural shifts, such as loss of aggression among non‐nestmates, increased hostility towards other species or changes in foraging activity. Finally, ecology‐related features can involve shifts in habitat or food preferences. Many of these traits facilitate easy dispersal and high competitiveness. When combined with polydomy, in which a single colony inhabits multiple nests, polygyny may lead to unicoloniality. Unicolonial populations form a network of interconnected nests among which workers move freely and lack aggression, despite having low genetic relatedness. In their extreme form, called supercolonies, they can reach extraordinary densities, span hundreds of kilometres and effectively outcompete other invertebrates. In ant invaders, some of these traits are linked to reduced genetic variability, or inbreeding or reduced nestmate discrimination capacity caused by their introduction.

Encountering some of these links was what Blumenfeld et al. (2022) expected when they surveyed *T. sessile* populations. Named the odorous house ant, this species native to North America is highly adaptable and found across many habitats, including cities. The authors knew from earlier research that the urban colonies are larger, polygynous and potentially polydomous, unlike most colonies in natural settings.

Using a large‐scale sampling and smartly integrating phylogeographical, population genetic, chemical and behavioural methods, Blumenfeld et al. disentangled the social structure of *T. sessile*. They confirmed with microsatellite markers that the colony structure is indeed flexible and linked to a habitat. The polygynous colonies were much more common in urban settings, while monogynous colonies were prevalent in natural habitats. Surprisingly, polydomy was relatively rare and detected only on a few occasions. And perhaps more remarkable is that polygyny has not only independently and repeatedly arisen under the urban settings on such a large scale but also that both types of colonies were consistently highly distinct genetically and chemically (in their cuticular hydrocarbons), confirming that habitat is a strong driver of divergence.

A similar polymorphism has been documented, for example, in European wood ants (*Formica*). While these nonurban species are often monogynous and monodomous, they can be polygynous and polydomous in highly fragmented environments, such as small forest patches in agricultural areas (Seifert et al., 2010). Polygyny leading to low intracolony relatedness, combined with polydomy, was proposed as one of the pathways for the formation of supercolonies. The idea is that they arise from the loss of nestmate recognition or through selection for reduced aggression.

What Blumenfeld et al. (2022) found in odorous house ants was different. The relatedness among workers in polygynous nests was high, including those with many queens. The authors further showed with behavioural tests that the levels of aggression were also high among different nests, confirming that they do not form large polydomous colonies in most cases. Their findings have important implications and suggest another pathway to polydomy and a shift in dispersal strategy. High relatedness within the polygynous colonies of *T. sessile* is probably a result of the adoption of daughter queens to the maternal colony. Instead of flying off and founding colonies independently, the daughter queens of *T. sessile* use budding—a dependent colony foundation with the help of the worker ants of the maternal colony. To prevent inbreeding, which this reproduction mode may lead to, *T. sessile* probably favours male‐biased dispersal to avoid sib‐mating. Such strategies were proposed by theoretical models and also documented in ants on islands or in patchy, isolated habitats. Whether this is a response to dispersal limitation, the high unpredictability of nesting sites or the patchiness of food sources in urban settings is a pending question.

By performing a detailed mapping of social and population structures as done by Blumenfeld et al., we can better assess the effect of ecological conditions on various life‐history traits. The authors placed their results into a broader perspective on how disturbance and urban environments can affect different taxa. They also propose reliable scenarios of how particular conditions in cities could lead to the specific traits and strategies encountered in urban odorous ant populations. Their work provides another excellent example of urban‐mediated shifts or adaptations and opens an array of questions to be addressed with follow‐up studies.

For example, genomic comparisons could map if there is selection towards specific sets of traits at the level of individuals. In the ant *Lasius niger*, workers in urban colonies had more diverse cytochrome gene families and DNA repair systems, while the olfactory systems were reduced (Konorov et al., 2017). These can be suitable preadaptations for exploiting urban environments or results of selection for pollutant resistance as seen in some other city‐dwelling species.

The substantial differentiation found among populations of *T. sessile* in urban vs. natural settings also deserves further attention. While another study of tiny acorn ant *Temnothorax nylanderi* did not reveal signatures of urbanization on population demography and structure (Khimoun et al., 2020), others have convincingly documented trait divergence and local adaptations in thermal tolerance between urban and rural populations of a related species (Martin et al., 2021). In *T. sessile*, we can look into the mechanisms reinforcing the urban and natural habitat barriers and the chances for human‐mediated dispersals. Robust markers should assess the population divergence among more habitats and spatial scales. Isolation by environment can regularly produce considerable population structuring; therefore, comparing the differentiation levels across diverse habitats would help us assess the contribution of urban‐specific factors to population divergence in *T. sessile*.

Finally, a macroevolutionary perspective may provide helpful insights into what traits are primary preadaptations for exploiting urban environments or becoming invasive. Ancestral shifts in preference to open and disturbed habitats in several South Pacific ants increased their macroevolutionary dispersal rates. These lineages then successfully colonized remote islands and broadened their distribution considerably, including giving rise to several invasive species (Matos‐Maraví, Clouse, et al., 2018a; Matos‐Maraví, Matzke, et al., 2018b). Comparative phylogenomic analyses of trait evolution among closely related species of *Tapinoma* and across unrelated clades with similar life features would help assess the contribution of different factors for succeeding in disturbed environments.

Organisms may respond to many different aspects of the urban environments, and these phenotypic and genotypic differences do not always imply adaptation (McDonnell & Hahs, 2015). Social insects are great models to study the environmental drivers and effects of different types of adaptive responses in which we can link individual‐level traits with the colony‐level and population‐level changes. Ultimately, they can help us reveal what a particular life history does to individuals and their social structure. Studies such as that of Blumenfeld et al. (2022) make great use of these large‐scale experimental settings called cities and provide deeper insights into the effects they have on plants and animals.

## FUNDING INFORMATION

CONACYT DICB 282471.

## ACKNOWLEDGEMENTS

None.

## CONFLICT OF INTERESTS

The author declare no conflict of interest.

## DATA AVAILABILITY STATEMENT

None.
